# New Appliances and Things Medical

**Published:** 1895-04-27

**Authors:** 


### NEW APPLIANCES AND THINGS MEDICAL.

[We shall be glad to receive, at our Office, 428, Strand, London, W.C., from the manufacturers, specimens of all new preparations and appliances which may be brought out from time to time.]

### L.G.B. CORROSIVE SUBLIMATE PELLETS.

(The Sanitas Company, Limited, Bethnal Green, London, E.)

Although other makers have placed on the market the Local Goverment Board's disinfectant in a tablet form, there cannot be any doubt that provided they are accurately made so as to contain a uniform quantity of mercuric chloride, they are the most handy form of disinfectant we have. Each pellet is supposed, when dissolved in a pint of water, to yield a blue solution containing one part corrosive sublimate in 1,000 parts of water. The Sanitas Company place their pellets n a tube fixed with a metal cap and marked poison in red letters. As each pellet is of large size and of deep violet colour they are not likely to be confounded with any other preparation; but as they are extremely poisonous, too great a care in keeping them out of the hands of uneducated persons and children cannot be taken, especially as they have somewhat the appearance of a sweetmeat. The weights, in grains, of three pellets taken haphazard out of the tube sent us for report were as follows:—

| | Weight of Pellet. | Weight of HgCl2 |
|---|---|---|
| 1. ... | 0·951 | 0·5576 |
| 2. ... | 0·899 | 0·5191 |
| 3. ... | 0·954 | 0·5766 |

It will thus be seen that the pellets are of very uniform weight and composition, and as one pint of water weighs 567 grains, they will each furnish an approximately 1·1000 solution when dissolved in water.

### LAURENT PERRIER COCA TONIC CHAMPAGNE.

(AGENTS: HERTZ AND COLLINGWOOD, 4, SUSSEX PLACE, LONDON, E.C.)

The preference exhibited in this country for champagnes without an appreciable amount of sugar seems warranted from a medical point of view, notwithstanding the fact that on the continent, and especially in Russia, our insular tastes in this direction are not followed. When, however, a dietetic wine is required there can be no doubt that an absence of saccharine matter is of extreme importance, and Messrs. Hertz and Collingwood have done well in securing a champagne practically free from glucose for the manufacture of their Coca Tonic wine. Our analysis of one of the sample bottles sent us for examination shows only 0·44 per cent. of dextrose and an entire absence of any sugar capable of inversion. We are therefore satisfied that this champagne is a naturally fermented wine made without the addition of syrup, and consequently free from added alcohol or sugar.

### CHOCOLATE SUCHARD, COCOA ESSENCE SUCHARD, SOLUBLE COCOA, SUCHARD.

(AGENTS, ALFRED BRAUEN, 38, HOLBORN VIADUCT, E.C.)

### BOISSELIER'S COCOAGENE.

(C. BARRY AND CO., FINSBURY, LONDON, E.C.)

Suchard's cocoa preparations are so well known on the Continent that it is surprising that they are not more frequently met with in this country. The samples sent to us for examination fully maintain the reputation of the manufacturing firm at Neuchatel, and our analysis confirms the statements made concerning them. The quantity of fat present in these samples was 24·11 per cent. in the soluble cocoa and 23·04 per cent. in the cocoa essence and chocolate, whilst the amount of sugar present was very small in the cocoas, and only 1·86 per cent. in the chocolate. For the full aromatic flavour of the cocoa nib they leave little to be desired. Boisselier's Cocoagene is a pure compressed cocoa extract made up into tablets of such a size that each tablet is suitable for making one cup of cocoa. Our analysis shows that this form of cocoa contains 6 07 per cent. of water, 8·02 per cent. of ash, which is somewhat too high, 22·17 per cent of fat, and 0·26 of sugar. The tablets seem to have been carefully prepared, and the cocoa is in a very fine state of division, and does not contain any trace of the shells of the cocoa nib. Cocoagene is manufactured by a patented process, and is guaranteed to be pure cocoa from which part of the fat has been extracted. The tablets are sold in cardboard boxes, containing eighteen, at such a price that three cups may be obtained at the expenditure of one penny.

### DR. THEINHARDT'S INFANTS' FOOD AND HYGIAMA.

(LONDON AGENTS, SORENSEN AND CO., 38, GREAT TOWER STREET, E.C.)

Dr. Theinhardt's preparations have already been much sought after in Germany where they were first brought out, and now that they are being introduced into this country we were pleased to receive samples from their London agents for examination and analysis. The idea underlying the two preparations is to limit the proportion of insoluble and useless material as far as possible and so produce a food stuff which shall contain the maximum amount of nutritious matter in a given weight. Dr. Theinhardt also seems to assume that starch is an undesirable food stuff and aims at the conversion of it into other carbohydrates which are soluble in water. This partial hydrolysis of the carbohydrates into readily assimilable forms renders the food suitable for persons of weak digestion as it makes the work of assimilation of the simplest character. The food for infants is claimed to be the only perfect substitute for mother's milk and to be not only highly nourishing but completely soluble and self-digesting. According to Dr. Gustav Abel's analysis, however, we note that there is 14.21 per cent. of carbohydrates which are not soluble, in cold water, and he finds only 80·87 per cent. of total soluble and assimilable nutritious matter. It is therefore evident that the statement of the proprietors has to be modified in this respect. Our own analysis confirms that of Dr. Abel, as we find that hot water extraction leaves 18·7 per cent. of insoluble matter, containing 4·34 per cent. of fat, thus leaving for starch and other insoluble constituents 14·36 per cent. When boiled with water for a long period the starch will, of course, become gelatinised. The food contains, according to our analysis, 10·9 per cent. of moisture, and this must be a variable quantity, seeing that the proprietors do not pack their product in tin or other moisture-proof boxes. The mineral matter amounted to 2·99 per cent., and contained about one-third its weight of phosphoric acid. The nitrogenous matter which forms a measure of the blood and flesh forming value of this preparation gave 2·05 per cent. of nitrogen, equivalent to 12·8 per cent. of albumenoids. The soluble carbohydrates showed the presence of maltose amounting to 22·58 per cent., and after inversion a further quantity of carbohydrate which reduced Fehling's solution was obtained. This, calculated to dextrin, gave 23·33 per cent., making a total of soluble heat-giving constituents of 45·91 per cent. It will be thus seen that this infants' food conforms very closely to König's standard, and is especially rich in soluble carbohydrates, fat, and bone-forming salts. As a dietetic it possesses a very high value, and if always of uniform composition can be relied upon as a satisfactory infant's food.

Dr. Theinhardt's Hygiama is a similar mixture of food-stuffs made from wheat and cocoa, which, when used in the proportion of two-thirds of an ounce to half a pint of milk, forms a highly nutritious beverage, which, according to the manufacturers, renews the vital powers, strengthens digestion, and restores the nervous system. From our analysis we can confidently recommend this preparation as containing valuable strengthening and nutritive ingredients. It is said, however, to be peptonised by a vegetable pepsin, but we are not informed what enzyme is employed. Some of the nitrogenous constituents are in a soluble form, but we have failed to detect any pe ptone. On previous occasions we have had to warn our readers against misleading statements of this character, and we hope that manufacturers will cease to use this term unless they can guarantee a definite percentage of peptone, *i.e.* of an albumenoid which is not precipitated by ammonium sulphate and gives the biuret reaction in the dialysed solution. Our analysis of Hygiama shows that it contains 3·28 per cent. of moisture, 3·02 of mineral matter rich in phosphates, 3·55 per cent. of nitrogen equivalent to 22·19 per cent. of albumenoids, 6·10 per cent. of fat, 11·5 per cent. of maltose, and 27·59 per cent. of invert matter calculated as dextrin. Hygiama is thus much richer in nitrogenous compounds and fat than the infants' food. When made into a beverage with milk it forms a solution not unlike cocoa, but differs from that made from the prepared cocoas in containing a different amount of starch and cane sugar.

